# Advanced nano-texture, optical bandgap, and Urbach energy analysis of NiO/Si heterojunctions

**DOI:** 10.1038/s41598-023-33713-y

**Published:** 2023-04-21

**Authors:** Laya Dejam, Jamshid Sabbaghzadeh, Atefeh Ghaderi, Shahram Solaymani, Robert S. Matos, Ștefan Țălu, Henrique D. da Fonseca Filho, Amir Hossein Sari, Hanieh Kiani, Amir Hossein Salehi shayegan, Mahdi Astani Doudaran

**Affiliations:** 1 Quantum Technologies Research Center (QTRC), Science and Research Branch, Islamic Azad University, Tehran, Iran; 2 Physics Department, West Tehran Branch, Islamic Azad University, Tehran, Iran; 3 Amazonian Materials Group, Physics Department, Federal University of Amapá-UNIFAP, Macapá, Amapá Brazil; 4 The Directorate of Research, Development and Innovation Management (DMCDI), Technical University of Cluj-Napoca, Cluj-Napoca, Cluj County Romania; 5 Laboratory of Synthesis of Nanomaterials and Nanoscopy, Physics Department, Federal University of Amazonas-UFAM, Manaus, Amazonas Brazil; 6 Physics Department, Science and Research Branch, Islamic Azad University, Tehran, Iran; 7 Mathematics Department, Faculty of Basic Science, Khatam-Ol-Anbia (PBU) University, Tehran, Iran

**Keywords:** Surfaces, interfaces and thin films, Materials for devices, Nanoscale materials, Optical physics

## Abstract

Due to the large number of industrial applications of transparent conductive oxides (TCOs), this study focuses on one of the most important metal oxides. The RF-magnetron sputtering method was used to fabricate NiO thin films on both quartz and silicon substrates at room temperature under flow of Argon and Oxygen. The sputtered samples were annealed in N_2_ atmosphere at 400, 500, and 600 °C for 2 hours. Using the AFM micrographs and WSXM 4.0 software, the basic surface parameters, including root mean square roughness, average roughness, kurtosis, skewness, etc., were computed. Advanced surface parameters were obtained by the Shannon entropy through a developed algorithm, and the power spectral density and fractal succolarity were extracted by related methods. Optical properties were studied using a transmittance spectrum to achieve the optical bandgap, absorption coefficient, Urbach energy, and other optical parameters. Photoluminescence properties also showed interesting results in accordance with optical properties. Finally, electrical characterizations and I–V measurements of the NiO/Si heterojunction device demonstrated that it can be used as a good diode device.

## Introduction

As an oxide of metals with high free carrier density, excellent electrical conductivity, and high optical transmittance in the UV–VIS-NIR spectrum transparent conductive oxides (TCOs) are introduced^1^. They have many applications depending on their electrical conductivity values. Currently, the most widely studied and commercially commonly used TCOs are ITO (Sn:In_2_O_3_), FTO (F:SnO_2_), and ZnO-based materials^2^, that all have n-type conductivity. Due to the widespread use of TCOs in fabrication of transparent p-n junctions and in organic solar cells, studying their p-type is very important^3^.

Among p-type semiconducting materials which are important technologically with the special band gap energy in the range of 3.6–4 eV, nickel oxide (NiO) should be considered in particular^4–6^ the p-type TCOs are very important and NiO thin films because of specific features like superior stability have attracted a great deal of attention recently. They have been used as antiferromagnetic material^7^, material for electro chromic display devices^8^, photovoltaic devices, electrochemical supercapacitors, heat reflectors, photo-electrochemical cell, solar cells, and many optoelectronic devices^9^ and functional layer material for chemical sensors^10^.

The properties of the nanoparticles and thin films make very interesting features compared to the bulk material properties^11^. Therefore, several techniques specially have been used for synthesis of thin film and nanostructures of NiO such as spray pyrolysis^12^, plasma enhanced chemical vapor deposition^13^ and reactive sputtering^10^. Among them, reactive sputtering has been used the most. The RF reactive magnetron sputtering, amongst variety of methods, is a simple process^14^ but highly effective method for preparing NiO thin films owing to its easier controllability of various parameters such as power^15^, oxygen partial pressure^16^, and substrate temperature^17^. NiO thin film can be prepared in various shapes such as nanowires and nanofibers^18^, nanotubes^19^, hollow hemispheres^20^, nanoflowers^21^, cactus-like structures^22^ and nanosheets^23^.

In general, in studies involving the morphology of surfaces, atomic force microscopy (AFM) is present, as it makes it possible to evaluate physical properties, with high precision, of surfaces for technological applications. Thus, due to its sensitivity and precision, the AFM technique provides morphological studies through the topographic maps that the scan generates, providing several morphological parameters^24–26^ and power spectrum density (PSD)^26^, facilitating the characterization of micro or nanoscale surfaces. The study of the distribution of topographic heights and their spatial complexity on surfaces of technological interest has provided great support in the optimization and fabrication of surfaces with improved physical properties, e.g., friction, adhesion, wettability, surface porosity, etc. Such analyzes allow an optimization of the manufacturing process of thin films and have been widely used in the study of the surface of thin films of technological interest. In our manuscript, it was observed that decreasing crystallite size generates rougher surfaces, however, with more homogeneous spatial patterns, indicating long-range correlations. This fact is important because other works have shown that surfaces with spatial patterns with more homogeneous distribution are less prone to failures, e.g., wear and cracks. In addition, it was verified, through advanced fractal and fractal parameters, that the roughest surfaces have more uniform spatial patterns and approximately ideal surface percolation, confirming the increase in topographic homogeneity according to the increase in annealing temperature.

In this work, NiO thin films were prepared by RF reactive sputtering method and the effect of annealing temperature on the structure and electrical properties were studied. We also studied statistical parameters related to the surfaces of these thin films using topographic images obtained by the AFM technique. It is worth mentioning that all the parameters presented in this work are in accordance with the international standard ISO 25178-2:2012. To complete the morphological study, we made use of two other fractal parameters, which were surface entropy and fractal succolarity. Therefore, our work is focused on the structural, 3D morphological and optical analysis of the films so that a complete analysis of the optical-morphological-structural relationship of the films can be obtained, which we believe to be of great importance for the optimization of the fabrication processes of these thin films.

## Materials and methods

### Thin films deposition

RF-magnetron sputtering system used to synthesis of NiO thin films on quartz and silicon substrates. The sputtering target was nickel metal in 99.99% purity. Before deposition process, the target was cleaned by pre-sputtering for 12 min. Substrates (10 × 20 mm^2^) were cleaned by ultrasonic waves in both of acetone and alcohol ambient respectively. The films were deposited at room temperature at base pressure of 2 × 10^–5^ mbar by rotary and turbo pumps while working pressure was fixed at 3 × 10^–3^ mbar by introducing argon (70%) and oxygen (30%). The best obtained RF power was set at 110 W. After that, annealing was started by N_2_ atmosphere at 400, 500, and 600 °C for 2 h with the rate of 10 °C/min then, they were cooled down to room temperature without any interference. Our goal was to obtain a diode as well as a transparent solar cell, which was achieved at temperatures of 400, 500, and 600 °C^27,28^. Deposition details are shown in Table 1.

**Table 1:** Optimal conditions for RF reactive sputtering experiments for the deposition of NiO thin films.

Sample	Substrate	Sputtering parameters				Thickness ± 5 (nm)	Annealing temperature (°C)
		Base pressure (mbar)	Working pressure (mbar)	Power (W)	Deposition time (min)		
#1	Si	2 × 10^−5^	3 × 10^−3^	110	100	200	–
#2	Quartz	2 × 10^−5^	3 × 10^−3^	110	100	200	–
#3	Quartz	2 × 10^−5^	3 × 10^−3^	110	100	200	400
#4	Quartz	2 × 10^−5^	3 × 10^−3^	110	100	200	500
#5	Quartz	2 × 10^−5^	3 × 10^−3^	110	100	200	600

After that, the as-deposited NiO films on silicon substrate (Si/NiO) were separately loaded to sputtering system to make front metal platinum contacts. During the process, a shadow mask with 1.0 mm diameter circular dots was used. After the front contact formation, whole back Si side of sample were coated with aluminum via sputtering system. The platinum and aluminum metals were used to obtained ohmic-type contact behavior. Hence, we aim to study only rectifying behavior of the p-NiO/n-Si heterojunction.

### Characterization

The DEKTAK3 profilometer measured the thickness of films. X-ray diffraction (XRD) was carried out on STOE-XRD diffractometer using Cu- K_α_ line (λ = 0.15406 nm) in the range of 10–90 degree. Atomic Force Microscopy (AFM) micrograph in contact mode was done by an Auto probe CP instrument from Park Scientific. Micrographs were done in a contact mode, having area of 1 × 1 µm^2^ and 256 × 256 pixel resolution each one. The Varian Cary-500 spectrophotometer was applied for optical properties also photoluminescence properties were examined by Cary Eclipse spectrometer 320 nm excitation wavelength were performed. The electrical calculations were done by current–voltage measurement by solar simulator (SIM-1030) and Palm Sense. The I-V curve was calculated under 1000 W/m^2^ of light source for Si/NiO heterojunction. All measurements were performed at room temperature.

### Morphological and Fractal analysis of the films surface microtexture

The surface parameters, such as Root Mean Square Roughness (Sq), Average Roughness (Sa), Kurtosis (Sku) and Skewness (Ssk), that were the basis for the sample’s morphology surface analysis, were in accordance with the ISO 25178-2: 2012 standard. These parameters were largely described in Refs.^29–31^. To compute those parameters, the WSXM 4.0 software was employed^32^. Furthermore, we also evaluated the discontinuity of the height distribution through topographic homogeneity, and this can be investigated through Shannon entropy. As no commercial software provides these measures, we obtained this parameter through an algorithm developed by Matos et al.^33^. So, according to Eq. (1), the Shannon entropy was used to calculate the surface entropy^34^

1 $${\mathrm{E}}^{(2)}=-{\sum }_{\mathrm{i}=1}^{\mathrm{N}}{\sum }_{\mathrm{j}=1}^{\mathrm{N}}{\mathrm{p}}_{\mathrm{ij}}\cdot {\mathrm{logp}}_{\mathrm{ij}}$$

where p_ij_ is the probability of having or not having outliers in terms of heights. Using Eq. (2), entropy was normalized in order to obtain uniform and non-uniform height distribution patterns:

2 $$\mathrm{E}=\frac{{\mathrm{E}}^{(2)}-{\mathrm{E}}_{\mathrm{min}}^{(2)}}{{\mathrm{E}}_{\mathrm{max}}^{(2)}-{\mathrm{E}}_{\mathrm{min}}^{(2)}}$$

in this equation, $E_{\max }^{(2)}$ is the surface with uniform minimum patterns and $E_{\min }^{(2)}$ is the non-uniform pattern surface. In this work, we calculated the $E_{\max }^{(2)}$ values that were represented by the symbol E.

The PSD of samples was also obtained, being calculated using the box counting method by the WSXM software, and from a linearized graph of the PSDs, we obtained the Hurst Coefficients (HC) of the spectra using Eq. (3):

3 $$HC=\frac{\gamma -2}{2}$$

Finally, the Fractal Succolarity (FS) was extracted using an algorithm developed in R language, as FS no commercial software provides this parameter. FS was obtained through Eq. (4)^35^:

4 $$\mathrm{FS}\left(\mathrm{T}\left(\mathrm{k}\right),\mathrm{dir}\right)=\frac{{\sum }_{\mathrm{k}=1}^{\mathrm{n}}{\mathrm{P}}_{0}\left(\mathrm{T}\left(\mathrm{k}\right)\right).\mathrm{PR}(\mathrm{T}\left(\mathrm{k}\right),{\mathrm{p}}_{\mathrm{c}})}{{\sum }_{\mathrm{k}=1}^{\mathrm{n}}\mathrm{PR}(\mathrm{T}\left(\mathrm{k}\right),{\mathrm{p}}_{\mathrm{c}}}$$

The dir is the water inlet direction, T(k) are equal sized boxes T(n), Po(T(k)) is the percentage occupancy, PR is the occupancy pressure, and pc is the position of the centroid (x, y) of pressure applied to the calculated box.

## Results and discussion

### Structural analysis

**Figure 1:**
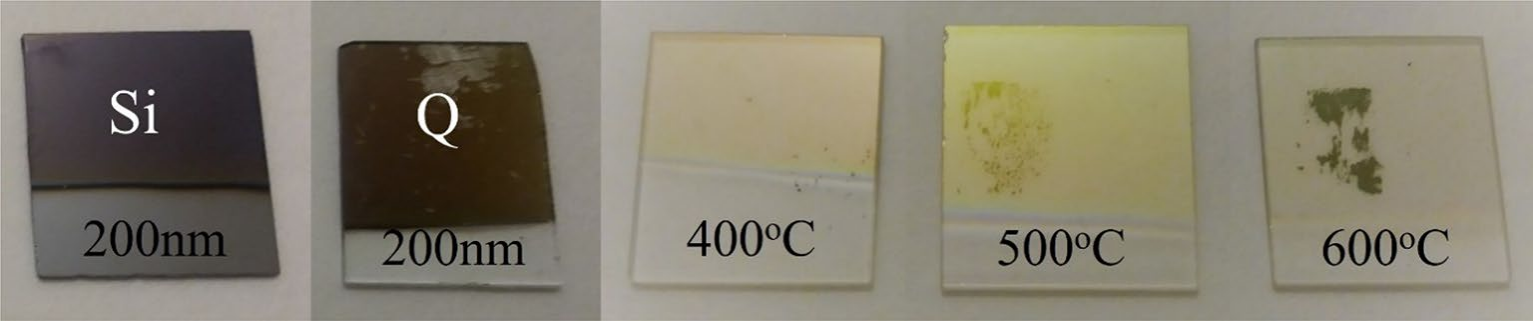
NiO thin films deposited on Si and Quartz.

As shown in Fig. 1, the NiO thin films became clearer as the annealing temperature increased which could be due to the stoichiometric changes of NiO thin films^27^. Due to the change of annealing temperature the reaction rate changes with the variation of the amount of nickel atoms.

Changes in stoichiometry and crystal structure of nickel oxide thin film due to annealing lead to changes in light scattering to the layers. In this way, with the increase of the annealing temperature, the quality of the crystalline structure has improved. Therefore, the scattering of radiant light is reduced, and the layer has changed color, so has become more transparent^27,36^.

The NiO nanoparticles, as synthesized through chemical means, exhibited an evolution of color from black to green with an increase in the annealing temperature, following the same trend as the mean size of the nanoparticles. The change in color from green to black for NiO nanoparticles is attributed to the presence of Ni vacancies (point defects). The colors of the NiO samples and EDS spectra confirm that the stoichiometry of chemically synthesized NiO nanoparticles decreases with decreasing particle size and that small nanoparticles (i.e., up to 14(3) nm) are highly non-stoichiometric^37^.

**Figure 2:**
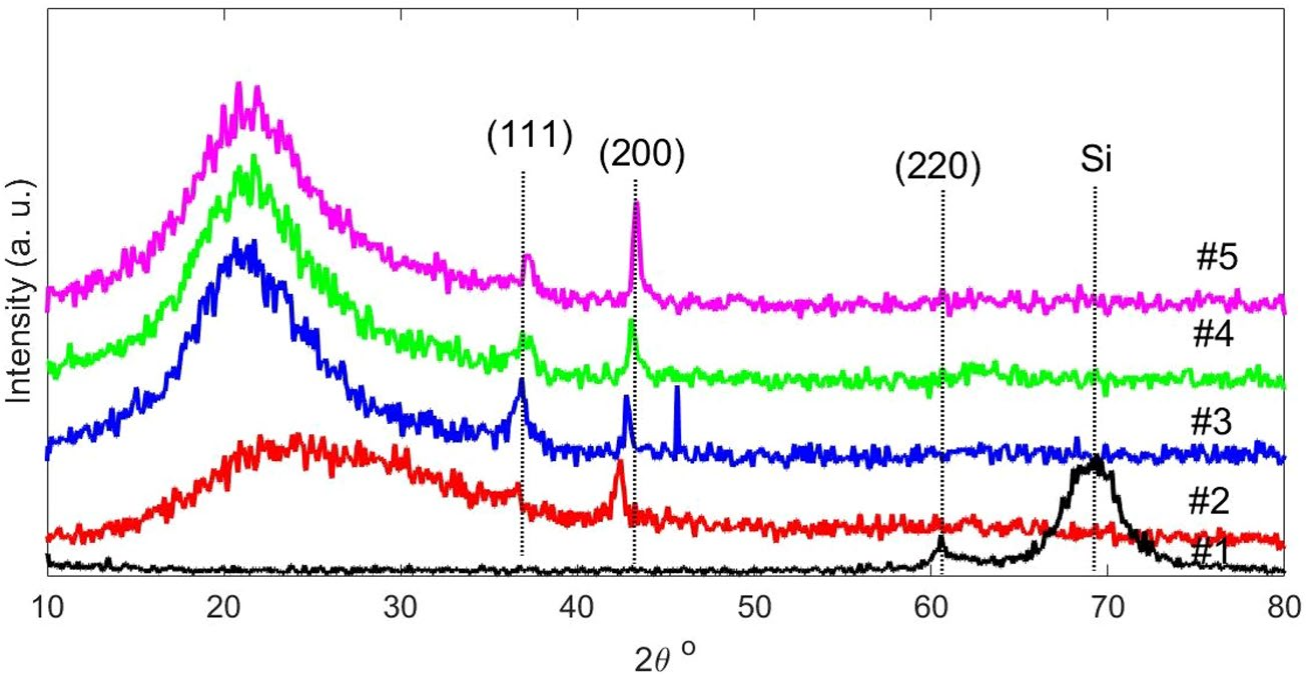
X-ray diffraction spectrum of NiO thin films with different annealing temperatures.

Figure 2 shows the XRD patterns of NiO thin films with different annealing temperatures. In the XRD pattern of NiO thin film two peaks appeared and got stronger in intensity as the annealing temperatures increased. Peaks are occurred at 36.61 and 42.40 degree which shows one degree displacement to higher degree in comparing with the JPDS card No. [01-078-0423]. These peaks are due to the cubic NiO structure. The one-degree displacement may be due to the non-stoichiometry NiO thin films, which is confirmed by the color of layers.

As it is clear in Fig. 2. The intensity of the peak at 42.4° (200) is much higher than the intensity of the peak at 36.61° (111). This could be due to the fact that the surface free energy of the (200) plane is lower than the (111) plane^36^. It is worth to mention that during the growth of NiO thin film, because of Joule heating effect the intensity of the peak due to the (111) plane is greater than the (200) plane^38^. Karpinski et al.^38^ shown that the preferred orientation peak at 42.2° is due to the (200) plane and its formation depends on the amount of oxygen which are present during the growth process.

Scherrer's formula was used to calculate the size of crystallite for film with thickness of 200 nm^37^. NiO lattice parameter is calculated by

5 $$a=\frac{\lambda }{2sin\theta }\sqrt{{h}^{2}+{k}^{2}+{l}^{2}}$$

when λ = 0.154 nm is the X-Ray wavelength, $\theta$ is diffraction angle and $h$, $k$, $l$ are Miller indices. The results are listed in Table 2.

**Table 2:** Result of calculations for XRD analysis of NiO thin films with 200 nm thickness on quartz substrate.

Annealing temperature (°C)	Substrate	Angle (2$\theta$) (°)	Lattice parameter (Å)	FWHM (°)	Crystallite size (nm)	Strain
–	Si	62.80	4.32	0.465	20.08	0.34
–	Quartz	42.40	4.26	0.020	42.05	0.02
400	Quartz	42.82	4.22	0.421	17.55	0.47
500	Quartz	43.07	4.19	0.442	16.72	0.49
600	Quartz	43.33	4.01	0.518	14.23	0.57

According to Table 2 for annealed samples at temperatures of 400, 500 and 600 °C and the same thickness of 200 nm, in NiO thin films, the crystallite size decreased with increasing annealing temperature as expected. In previous research, the crystallite size has increased with increasing temperature^39^. The NiO thin film grown on quartz substrate compared to silicon one shows an increase in the lattice parameter but decrease in the crystallite size.

The NiO thin films grown on the silicon, the crystal plane is changed, and the crystallite size is much smaller than in the quartz substrate. We assume that the adhesion of NiO to the silicon substrate is much greater than that of quartz. Because NiO atoms that disperse on the surface of silicon, so they have more adhesion to Si than similar atoms, much smaller crystallites are formed, but in quartz substrates, the surface adhesion of NiO to similar atoms is much higher and many crystallites larger is formed.

By annealing at a temperature of 400 °C, there are two main peaks related to crystal planes (111) and (200) in a thin film of NiO. When increases annealing temperature the crystal plane (111) decreases and (200) peaks increase, A change in crystallite size was confirmed according by the optical analysis of the thin films, for example, the band gap of the thin films has increased with increasing temperature.

### Morphological and spatial analysis

**Figure 3:**
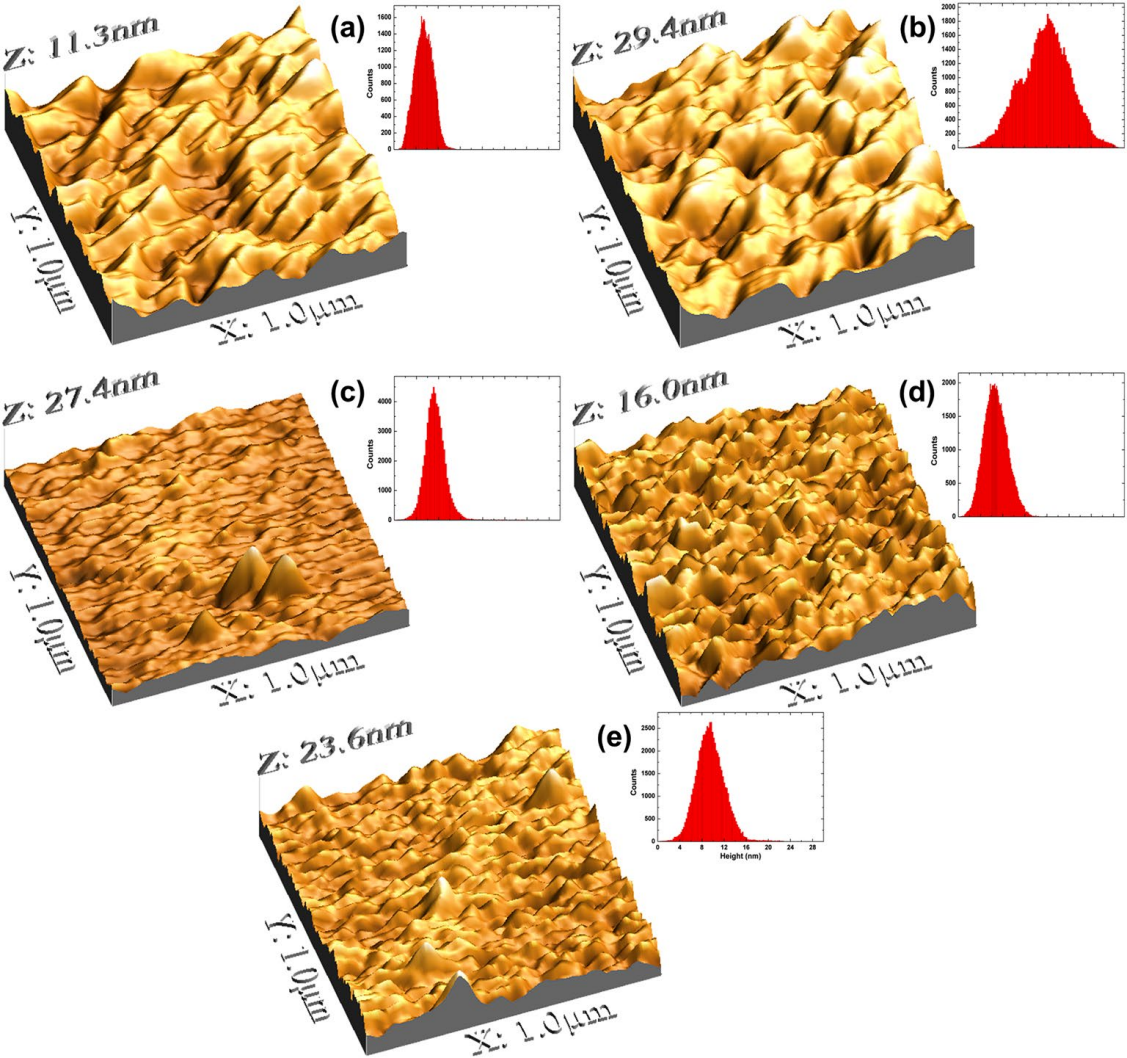
Representative 3-D AFM micrographs of (**a**) #1, (**b**) #2, (**c**) #3, (**d**) #4, and (**e**) #5. The insets represent the height distribution of each thin film.

The surface morphology of NiO thin films deposited on Si and quartz substrates is shown in Fig. 3. The as deposited NiO thin films onto Si (Fig. 3a) and quartz (Fig. 3b) reveal spatial patterns with different characteristics. The height parameters of the film deposited on Si have lower values, whereas higher values are observed for the film deposited on quartz substrate. This suggests that there is a characteristic morphology for every type of substrates. Such behavior is associated with the formation of crystals with different crystallite sizes, which is in perfect agreement with the XRD analyses (Table 2). Moreover, the thermal treatment imposed on as deposited films promoted a driving force capable of modifying the 3D spatial patterns, with evolution of the morphological aspects from 400 to 600 °C. The film treated at 400 °C (Fig. 3c) exhibits an irregular morphology with the presence of regions with discrepant rough peaks, whose formation may be due to the beginning of thinning. The grain thinning dictated the formation of fine and sharper rough peaks for #4 (Fig. 3d), whose grouping led to the formation of a surface with rough peaks of different characteristics in #5 (Fig. 3e). In this regard, the grain thinning due to temperature increase occurred due to crystallite size decrease, as observed in the XRD analysis.

**Figure 4:**
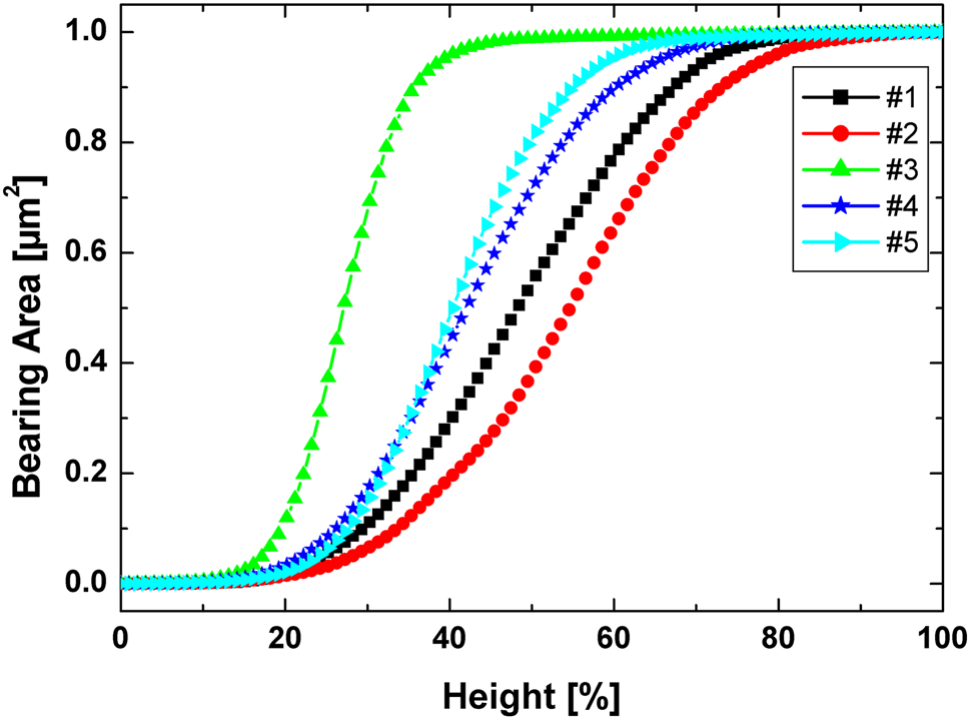
Abbott–Firestone curves of the samples.

The height-based parameters showed in Table 3 confirm that the substrate type and annealing temperature promote an evolution of the topographic spatial patterns of NiO thin films. As can be seen, the NiO thin film deposed onto quartz (#2) displays a rougher surface compared to Si (#1), which was observed for both root mean square roughness (Sq) and average roughness (Sa). In addition, the topographical height distribution for #2 has a slight right-skewing (skewness (Ssk) < 0), while for #1 it is skewed to the left (Ssk > 0)^40^. Despite this, both distributions exhibit a value of Ssk ~ 0 revealing that the distributions are almost symmetric (Table 3). Additionally, the #2 as prepared thin film exposes a height distribution with quasi platykurtic behavior (kurtosis (Sku) ~ 3)^31,41^, which is well supported by the height distribution displayed in the inset of the Fig. 3b. Furthermore, the Abbott-Firestone curves (AFC’s) displayed in Fig. 4 show that the AFC of #1 quickly approaches its maximum, while for #2 the increase is slower, confirming that the height distribution of the film deposited on quartz substrate is more centralized^42^.

**Table 3:** Relevant roughness and height parameters for the analysed samples from AFM images.

Parameter	Unit	#1	#2	#3	#4	#5
Sq	[nm]	1.63	4.47	2.16	2.21	2.63
Sa	[nm]	1.33	3.57	1.48	1.69	2.03
Ssk	[–]	0.05	− 0.12	2.17	0.23	0.50
Sku	[–]	2.65	2.84	16.5	2.95	4.47

On the other hand, the thermal treatment of NiO films deposited on quartz substrate produced surfaces with less roughness compared to the as deposited film (#2), which was also observed for both Sq and Sa parameters (Table 3). However, the temperature evolution provides surfaces with greater roughness from 400 °C, explicitly, 2.16 nm (#3), 2.21 nm (#4), and 2.63 nm (#5) (Table 3), which is ascribed to the reorganization of the fine grains that form the surface microtexture of the films. The Abbott-Firestone curves for #3, #4, and #5 (Fig. 4) reveal that the intermediate annealing temperature (500 °C) promotes the formation of a more centralized height distribution. This phenomenon is mathematically supported by the values of Ssk and Sku, as the sample #4 was the only one to promote the formation of a surface with a quasi-symmetrical (Ssk → 0) and quasi-platykurtic (Sku ~ 3) height distribution (Table 3). The mechanism behind the formation of leptokurtic surfaces (Sku > 3) of #3 and #5 may be associated with a strongly anisotropic behavior linked to the organization of NiO grains along the film.

**Figure 5:**
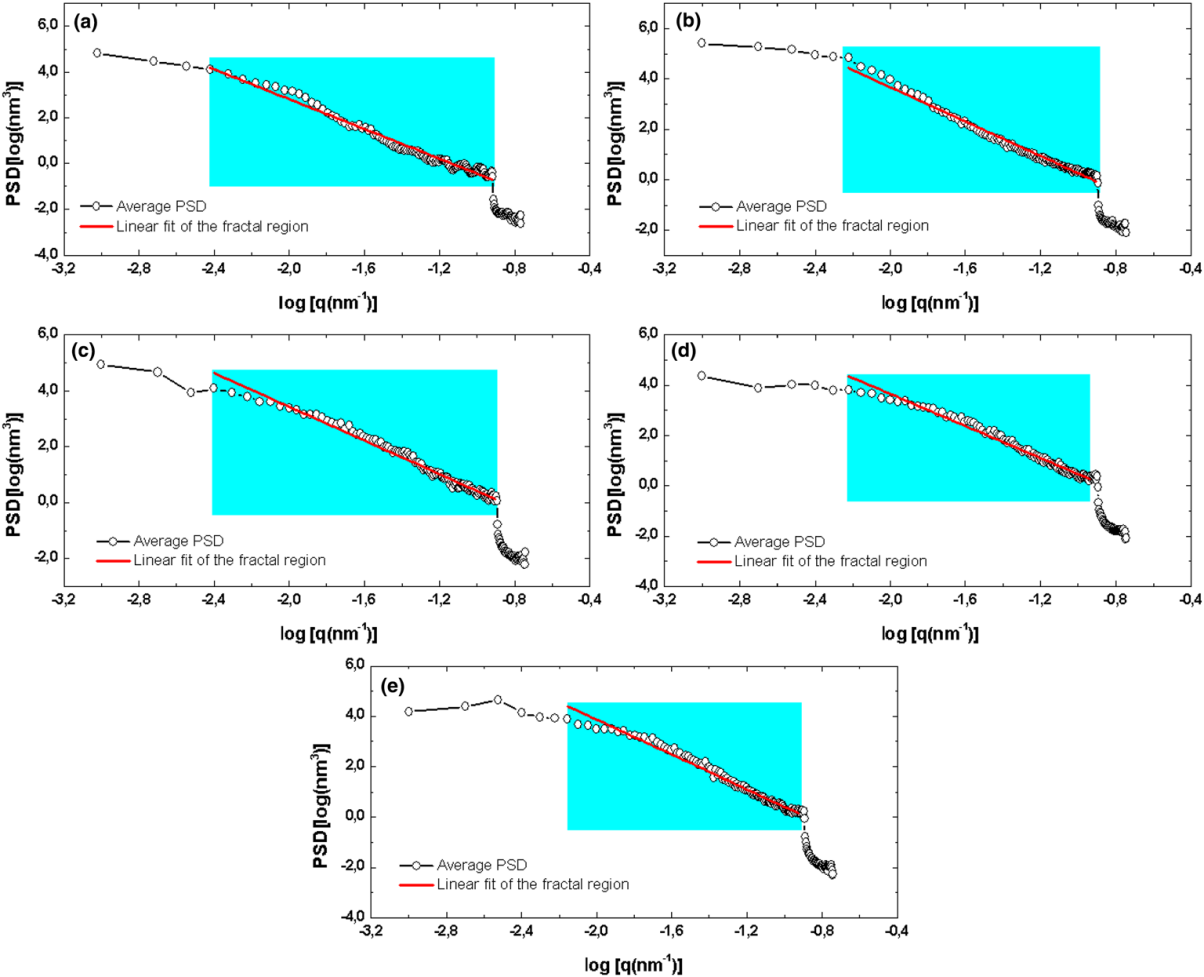
Average PSD and linear fit of the fractal region of the PSD spectra of (**a**) #1, (**b**) #2, (**c**) #3, (**d**) #4, and (**e**) #5.

The investigation of the surface microtexture of the films was based on the analysis of their fractal behavior, whose results are summarized in Table 4. In this regard, the as prepared thin films expose fractal dimensions (FD) of 2.27 (#1) and 2.26 (#2), indicating similar spatial complexity. However, the evolution of the surface microtexture of the annealed films reveals an increase of the spatial complexity from 400 to 600 °C. Such behavior proves that the thinning of the grains due to the thermal treatment promotes the formation of surfaces with more long-range spatial correlations compared to the as prepared thin films. The high spatial complexity found for the sample annealed at 600 °C (FD = 2.41) shows that its topographic irregularities create a multiscale roughness that favors large effective areas of contact over the surface^42^. The fractal behavior of the samples is also well supported by the PSD analysis shown in Fig. 5, which indicate that all films have 3D spatial patterns with a self-affine trend. The lines used to estimate the Hurst coefficient (HC) display a suitable adjustment to the experimental data. All samples displayed HC > 5, indicating that their height distributions are homogeneous and with probability > 50% of repetition of the height values^43^. As can also be seen, the value of HC increases from 0.511 to 0.728, showing that the annealing temperature plays a critical role on the formation of topographic height distributions more homogeneous. Therefore, the decrease in crystallite size promoted by the increase in annealing temperature dictated the thinning of the grains to generate rougher surfaces with more homogeneous 3D spatial patterns.

**Table 4:** Fractal parameters of the samples.

Parameter	Sample				
	#1	#2	#3	#4	#5
FD	2.27	2.26	2.29	2.36	2.41
HC	0.631	0.709	0.511	0.594	0.728
FS	0.359	0.470	0.406	0.443	0.448
E	0.972	0.997	0.991	0.999	1

Furthermore, the percolative analysis of the surface microtexture of the films shows that the as prepared films display different characteristics. The NiO film deposited onto Si substrate (#1) exhibited a less percolable surface, as the fractal succolarity (FS) was lower than for the film deposited onto quartz substrate (#2). The FS of the annealed samples increase of 400 to 600 ºC, showing that the homogenization of the topographical height distribution promotes more percolatable surfaces. It is worth noting that the FS value for #2 and #5 was ~ 0.5, which is considered an ideal surface percolation value^44^, proving that the as prepared #2 and the annealed film (#5) have the more homogenous surface microtextures. Likewise, the #2 sample presented a topographical uniformity greater than #1, which is supported by its higher topographical entropy value (E) (0.997). In addition, as a result of the increased homogeneity of the topographic height distributions from #3 to #5, an increase of the topographic uniformity was also observed after the heat treatment imposed. As can be seen, the sample #5 have a perfectly uniform topographical height distribution (E = 1)^33,45^. Thus, the annealing temperature increases the topographic roughness and spatial complexity of NiO thin films deposited onto quartz substrate and dictates the formation of more homogeneous and uniform surfaces.

### Optical analysis

**Figure 6:**
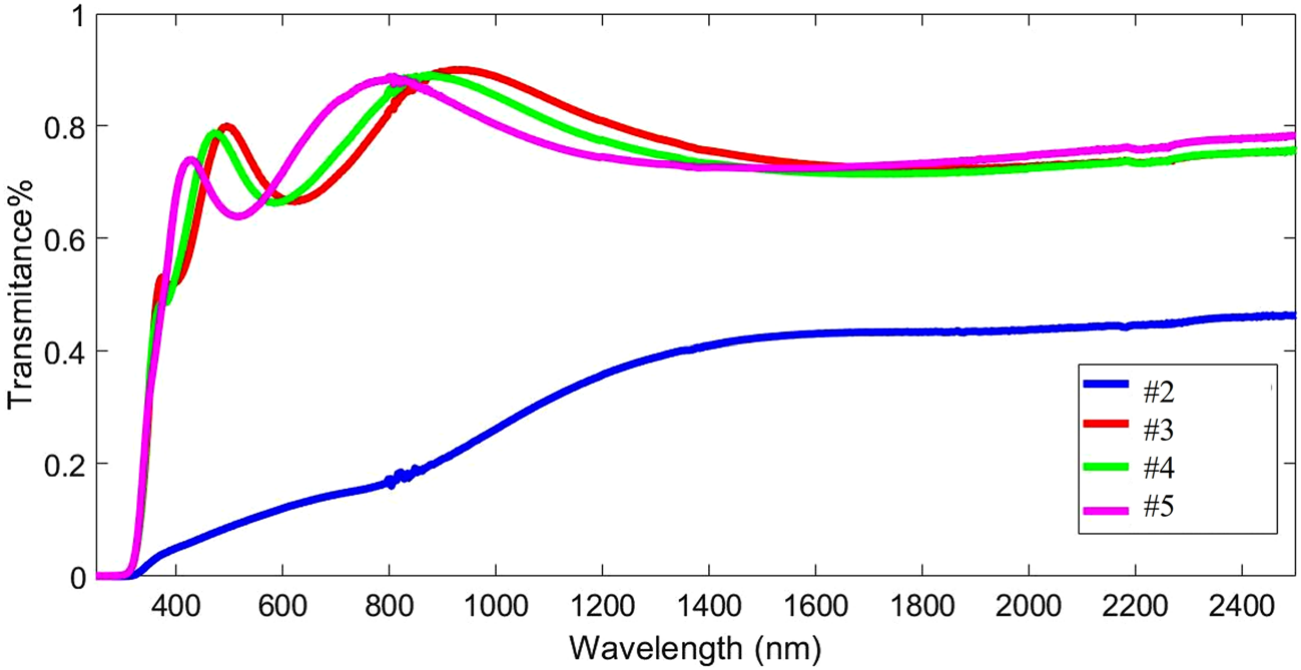
The transmittance spectra of prepared NiO thin films on quartz substrate with different annealing temperatures.

Figure 6 shows the transmittance spectrum of 200 nm thick NiO thin films on Si and quartz substrates. Annealing has increased the transmittance of the thin films from 30% to about 70%. This means that annealing has made the nickel oxide layers more transparent, but with changes in temperature from 400 to 600 °C, no significant changes in the transparency of the thin films were observed. That is, by annealing at 400 °C, the 200 nm thin film becomes transparent, and the higher temperature only causes the edge of absorption to change slightly.

In fact, higher temperatures of annealing have changed the crystal quality of the thin films or their surface uniformity. The structure of the wave spectrum in the transmittance spectrum of the annealed thin films shows that the interference and surface reflections of these films have created constructive and destructive interferences that the transmittance spectrum is in the form of a wave with valleys and peaks. But we do not see such a spectrum in unannealed samples.

**Figure 7:**
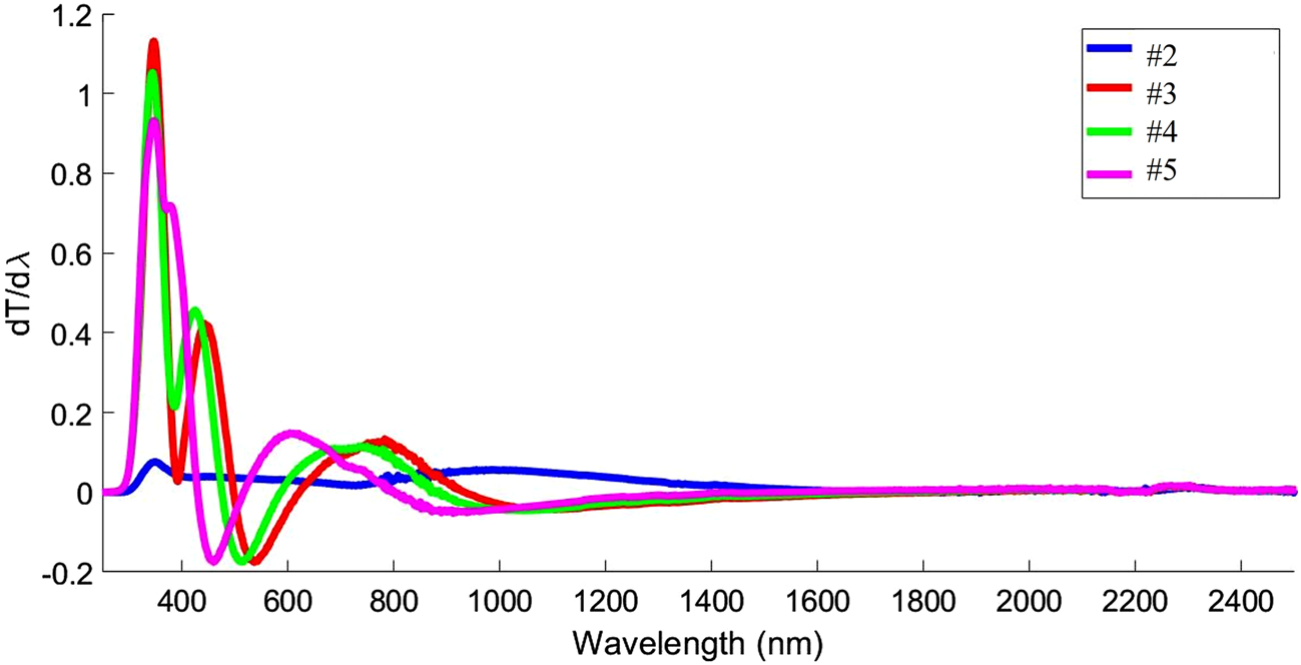
dT/dλ of NiO thin films on quartz substrate with different annealing temperatures.

The first derivative, dT/dλ, applied to investigation on transmission variations versus photon wavelength, shown in Fig. 7 with a maximum peak (λg) accordance with gap energy Eg = hc/λg. We know that for an ideal compound, zero transmission can be seen for wavelengths less than λ_Eg_^46^, so here, these peaks do not show an ideal compound. According to the values of dT/dλ and optical bandgap of NiO thin film, the optical bandgap of annealed samples has shifted to higher wavelengths or less energies. Direct relation of this parameter with three factors of absorption edge, donor carrier concentration, and impurity energy levels has been known. Shift of absorption edge to lower energies and consequently decreasing the band gap is due to decrease in impurity energy levels^47^.

**Figure 8:**
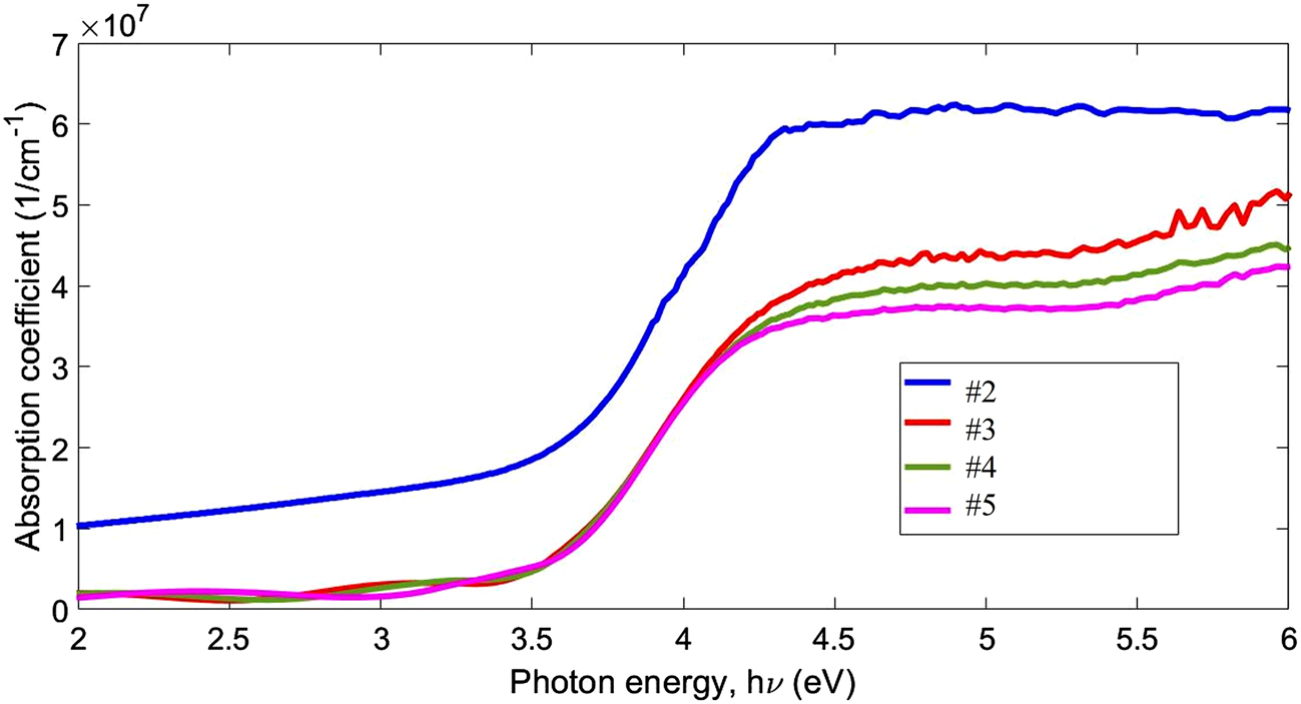
Adsorption coefficient of NiO thin films on quartz substrate with different annealing temperatures.

In the case of calculating the absorption coefficient by transmission spectra, the below equation was used to plotting α is plotted versus photon energy (Fig. 8). There, d is the thickness of films.

6 $$\alpha = {1}/{\text{d Ln }}\left( {{1}/{\text{T}}} \right)$$

The absorption coefficient of the annealed NiO films shows that with increasing the annealing temperature, the absorption edge has not changed much and only with increasing temperature, the adsorption edge has become slightly softer.

**Figure 9:**
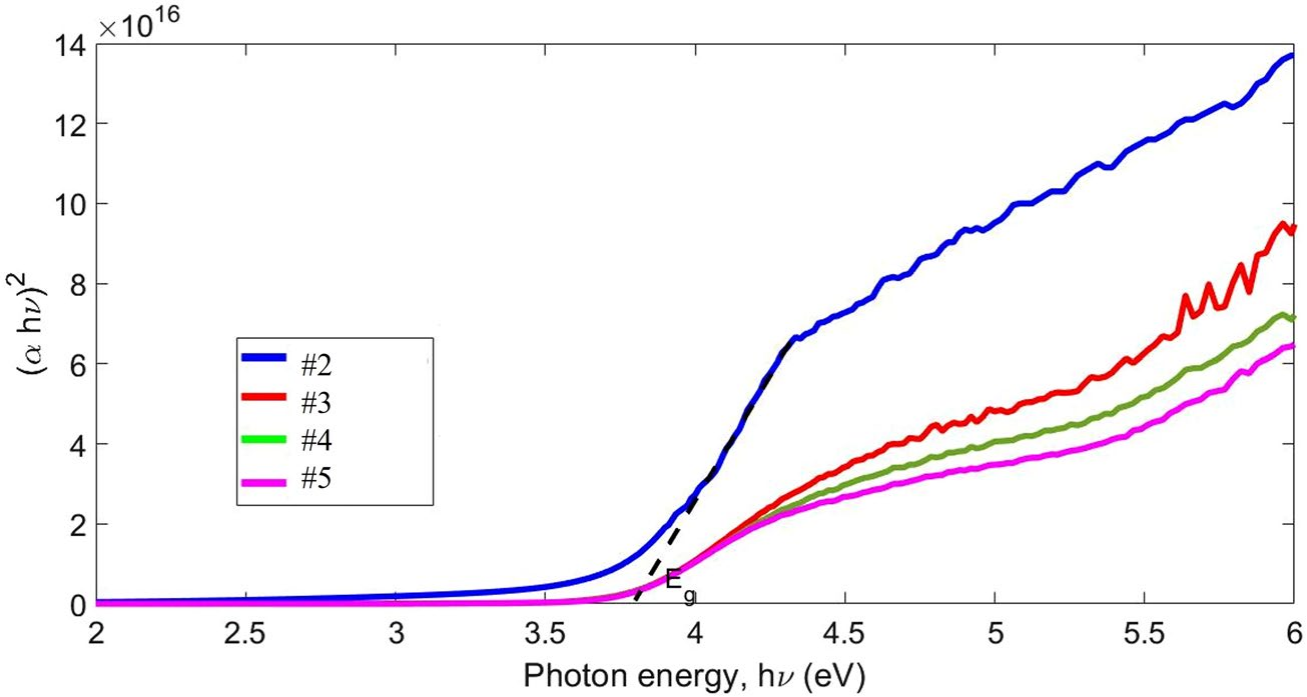
Determination of optical gap band of NiO thin films on quartz substrate with different annealing temperatures.

Applying the well-known Tauc relation in the appendix S1 (Part A), which has been described by Ilkhani, et al.^47^ in details and considering indirect allowed transition accordingly, the optical bandgap values of the NiO thin films were calculated (Fig. 9).

The calculated bandgap values are exhibited in Table 5. As the annealing temperature increased from 400 to 600 °C, the bandgap of the thin films decreased, which was to be expected due to the NiO X-ray diffraction spectrum, which became crystallized as the annealing temperature increased. Because by increasing annealing temperature the quality of crystallites increased so the localized states and traps in the thin films decreased then the bandgap decreases. Calculating the Urbach energy (E_u_) using Eq. (7) shows that the width of the traps decreases with increasing annealing temperature and causes the bandgaps to decrease.

7 $$\alpha = \alpha_{0} \exp (\frac{h\nu }{{E_{u} }})$$

**Table 5:** Bandgap calculations and Urbach energy of NiO thin films.

Sample	E_g_ (direct) [eV]	E_u_ [meV]
#1	3.75	641
#2	3.80	597
#3	3.75	312
#4	3.70	296
#5	3.60	342

Width of localized states gives E_u_, it means that in diagram of Lnα versus photon energy is the slope of line which has been summarized in Table 5.

In Fig. 9, the diagram (αhν)^2^ is plotted in terms of E to calculate the bandgap values of the annealed NiO thin films, and the values obtained are given in Table 6.

**Table 6:** The I–V parameters of n-Si/p-NiO heterojunction structure and the related literature data.

	Standard (I–V) method			Cheung–Cheung’s method		
	n	ϕ_b_ (eV)	I_S_ (A)	n	ϕ_b_ (eV)	R_s_ (MΩ)
Our work	2.86	0.48	1.52 × 10^−6^	3.05	0.54	0.11
Ref.^37^	2.39	0.85	–	3.77	0.45	0.19
Ref.^38^	2.29	0.82	2.59nA	–	–	–
Ref.^39^	2.26	0.79	–	3.27	0.36	0.97
Ref.^40^	3.7	0.81	–	–	–	–

*n* ideality factor, *ϕ*_*b*_ barrier height value, *I*_*S*_ saturation current, *R*_*s*_ series resistance.

By raising the annealing temperature, the quality of the thin films according to the XRD spectrum improves, and the bandgap of the thin films confirms this, because the bandgap values decrease with increasing temperature. The more crystalline the layers, the lower the bandgap values^48^. The Urbach energy values of the NiO thin films also show that increasing the temperature increases the Urbach energy and the localized states in the bandgap, which means that we have traps in the valance band that reduce the bandgap.

The Kubelka–Munk theory was used to transform the reflectance of thin films into a Kubelka–Munk function (F (R)) with Eqs. (8) and (9)^49,50^.

8 $$F\left(R\right)=\frac{{(1-R)}^{2}}{2R}$$

9 $$\alpha =\frac{F(R)}{t}$$

*R* is the reflectance of NiO thin film as a function of wavelength and t is thickness of NiO thin film.

**Figure 10:**
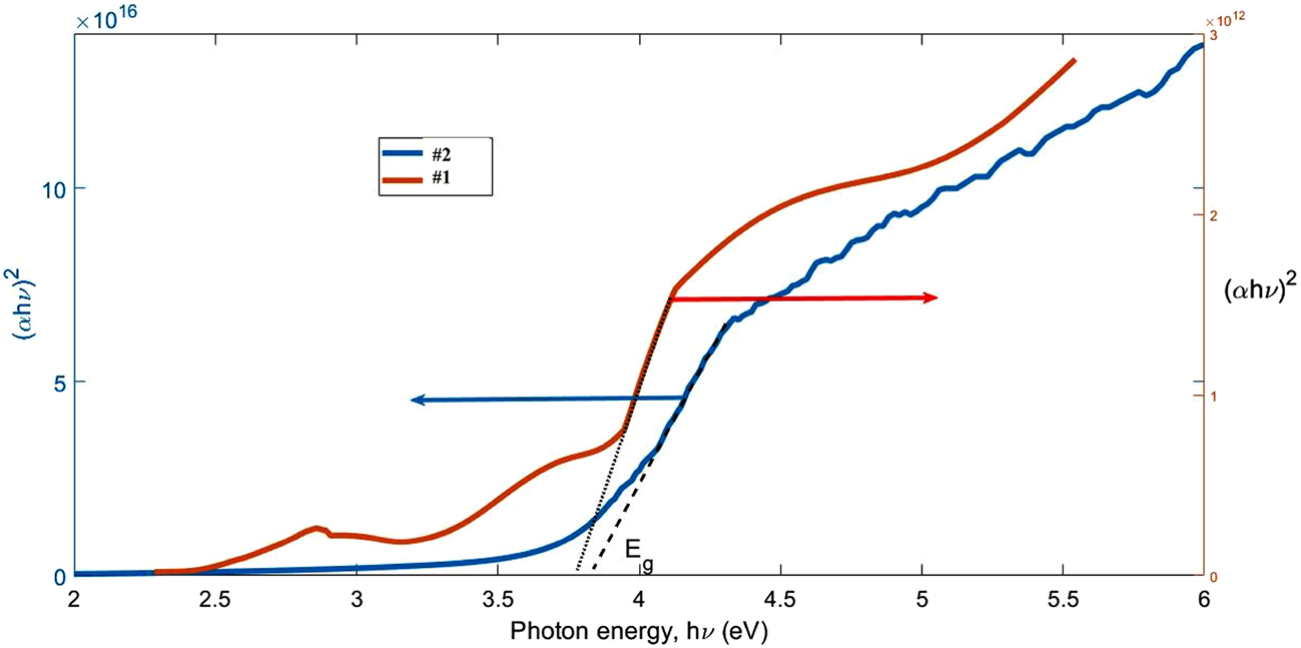
Determination of optical gap band of NiO thin films on silicon (#1) and quartz (#2) substrates.

To evaluate the band gap of Si/NiO thin film associated to their direct allowed transition and Tauc relation, (αhν)^2^ vs. hν is presented in Fig. 10. The evaluated band gap of Si/NiO and Q/NiO were measured from the linear line hν–intercept value. The band gap values are more near each other since substrate has a little effect on NiO band gaps.

The improvement of crystal microstructures reduces the scattering caused by defects. In our samples, the increase in the transmission of nickel oxide layers occurs with the increase of the annealing temperature and the improvement of the microstructure.

Photoluminescence (PL) spectroscopy is a powerful tool to characterize the optical quality of semiconductor metal oxides as PL intensity can be correlated directly with the defect densities. Therefore, the PL spectra of such metal oxides are strongly affected by cationic/anionic vacancies. This can give insight into the charge excitation, electronic structure, and defect states of oxides^37^.

On the other hand, in the PL spectrum, the intensity of the emission spectrum of nanoparticles has increased with the increase of the annealing temperature due to the decrease in the size of the nanoparticles. This reduction in particle size and smoothness of the surface has increased the transmission and intensity of the emission spectrum^51^.

**Figure 11:**
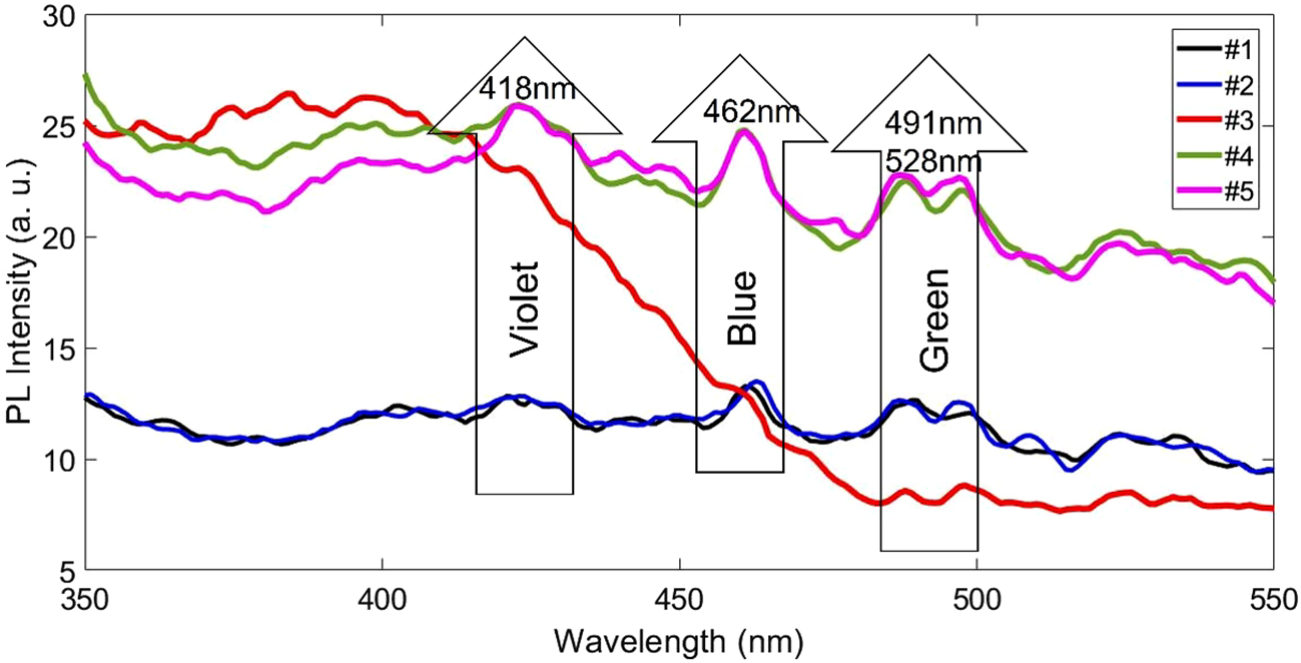
The PL spectra of as deposited and annealed NiO thin films.

**Figure 12:**
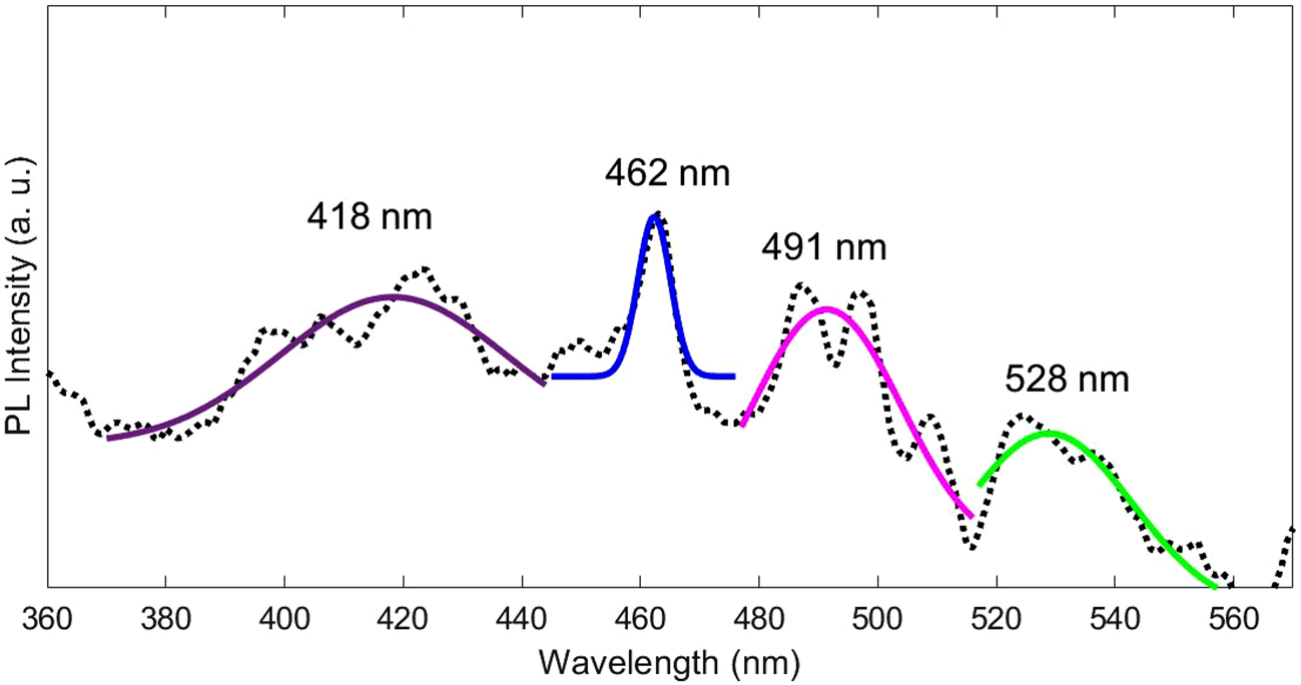
The PL spectra of Si/NiO heterojunction with gaussian fitting of four emissions.

Two categories of near band edge (NBE) UV emission and deep level (DL) defect related visible emission are considered for the PL emission of metal oxide nanostructures. The direct recombination of excitons through an exciton-exciton scattering usually is related to UV emission while the radiative recombination of a photo-generated hole with an electron occupying the oxygen vacancy is commonly origination of visible emission^52^. Figure 11 presents the PL spectrum recorded with an excitation wavelength of 320 nm at room temperature which is consists of 4 dominant peaks at 418, 462, 491 and 528 nm. Also, for more careful examinations of the peaks, Fit Gaussian for each of them is plotted in Fig. 12. We can propose that the emission peaks in the visible region is due to excitonic PL process that the non-radiative transition of excited electrons from bottom of conduction band to different sub-bands occurs then subsequent radiative transition from sub-band to top of VB take place. Vacancies, interstitial and defects of material make this excitonic PL. The violet emission peak at 418 nm, the blue emission peak at 462 nm and the green emission peaks at 491 and 528 nm can be considered as band-edge free excitons, defects induced by nickel vacancies or excess oxygen, and bound excitons respectively^52^. Consuming the blue emission peak at 462 nm originated from the intrinsic defect states, such as Ni and O interstitials or vacancies is not far from the mind. The PL spectra of NiO on silicon and quartz substrates have the same intensity, but after annealing, the intensity of peaks has increased, which is due to the decrease in the size of particles.

**Figure 13:**
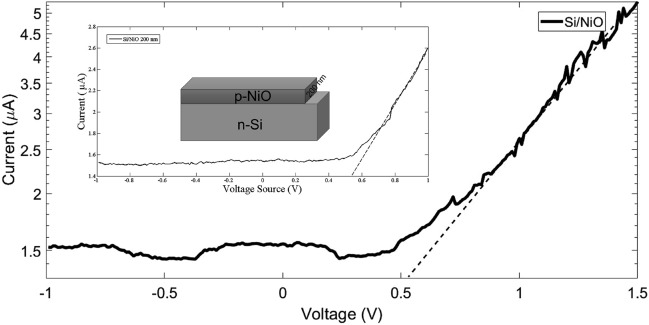
Current–voltage diagram for NiO thin film grown on silicon.

In Fig. 13, the current diagram is shown as a function of voltage, the NiO-p/Si–n structure exhibits the same behavior as the diode structure. It can also be seen that the current at linear voltages has changed linearly. At low voltages, the current changes linearly. At the scale and range of linear changes in current, the on-turn voltage for diode can be obtained. It should be noted that low turn-on voltage is much better to reduce drop-on voltage of diode and a higher output voltage can be designed for the rectifier. On a linear scale in current-based voltage curves the drop-on voltage of Si/NiO diode obtained 0.55 V.

**Figure 14:**
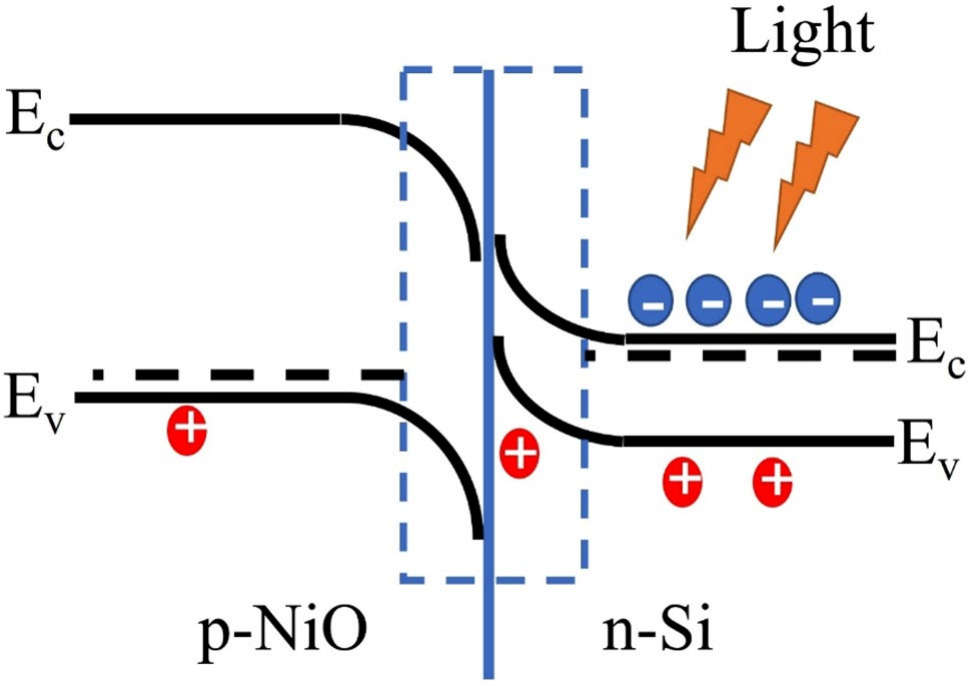
Schematic representations of the band energy and charge transfer for an n-Si/p-NiO heterojunction under light illumination.

**Figure 15:**
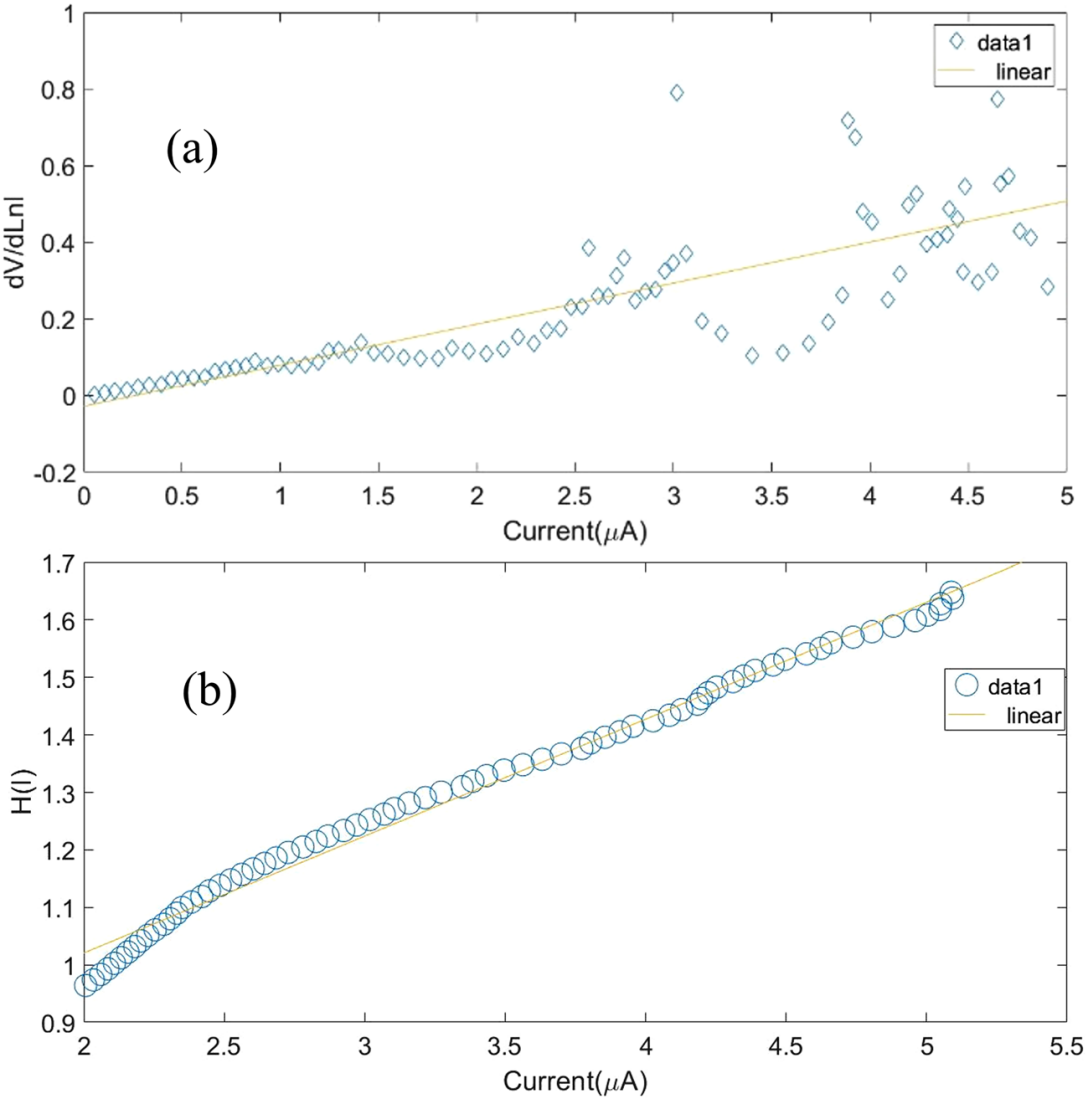
(**a**) dV/d(lnI)-I and (**b**) H(I)-I plots of NiO/Si heterojunction device at 300 K.

As is shown in Fig. 13 which is accordance with obtained data at room temperature and schematically in Fig. 14, I–V measurements was applied to study electrical parameters of NiO/Si heterojunction device. The heterojunction device presented a rectification behavior. In order to I–V characteristics we used a series of well-known equations^53^ that we refrained from presenting here to avoid the length of article while all the contents in detail have been brought in the appendix S1 (Part B). All I–V characteristics parameters of NiO/Si heterojunction device are summarized in Table 6. We compared our findings with literatures. The ideality factor of NiO/Si heterojunction device is 2.88, which is clearly greater than an ideal value (n = 1). It could be because of effect of surface state or maybe due to thin oxide layer at the interface of heterojunction^54^. Many Refs. give results on barrier height^55–58^, while the lowest φ_b_ is calculated in our study. We also determined series resistance and other electrical parameters based on Cheung–Cheung’s method^59^ which has been introduced completely in the appendix S1 (Part C). In this method the higher bias voltage region can be obtained using the slope of graph of Fig. 15. These results presented in Table 6 too. Here, the calculated value for ideality factor is 3.05 accordance with Yilmaz et al.^55^. Also, series resistance values confirm each other, and are lower than ones reported by Yilmaz et al.^55^. In the case of barrier height value, we calculated in our work and compared with other Refs. from the standard I–V method which is listed in Table 6.

Overall, the agreement of our device parameters with the Refs.^37–40^ is acceptable. Also, the change in diode parameters was related to the structural and morphological properties of prepared thin films. If the ideality factor is greater than 2, it may be originated from multi-level recombination channels because of more defects of junction’s interface it means that optimization of growth process can be considered as an effective way to increase the interface properties.

## Conclusions

The NiO thin films with 200 nm thickness were grown on Si and quartz substrates by RF reactive sputtering then annealed in 400, 500 and 600 °C. The structure and electrical properties of the prepared thin films were used to modify and classify the NiO films according to their applications.

The NiO thin films have a cubic crystal structure with (111) and (200) planes, and the crystallite sizes decreased with increasing annealing temperature. The comparison of crystalline structure and morphology properties showed that the type of substrate dictates the formation of surfaces with different vertical growth dynamics also the grain thinning under annealing temperature increase when crystallite size decrease. The Abbott-Firestone curves confirms that the height distribution of film deposited on quartz substrate is more centralized. Also, the fractal behavior of samples is also well supported by the PSD analysis. On the other hand, the percolative analysis of surface microtexture of films shows that the as prepared films display different characteristics. In the case of the annealing temperature study, we found that this parameter increases the topographic roughness and spatial complexity of films deposited onto quartz substrate and determines the formation of more homogeneous and uniform surfaces. The NiO thin films were dark and became transparent after annealing with 85% transmittance and their optical bandgap was between 3.60 and 3.80 eV. The ideality factor by Cheung–Cheung’s method was 3.05 and the barrier potential was larger than the standard method. Also, it was in good agreement with previous studies.

## Supplementary Information

Supplementary Information.

## Data Availability

The datasets used and/or analyzed during the current study available from the corresponding author on reasonable request.
