# Supplementary material for: Advanced nano-texture, optical bandgap, and Urbach energy analysis of NiO/Si heterojunctions
Source: Sci Rep. 2023 Apr 21;13:6518. doi: 10.1038/s41598-023-33713-y (PMC10121669; doi:10.1038/s41598-023-33713-y)
Supplement: Supplementary file 1 — Supplementary Information. [file 41598_2023_33713_MOESM1_ESM.docx]

**Advanced Nano-texture, Optical Bandgap and Urbach Energy Analysis of NiO/Si Heterojunction**

Laya Dejam^1,2^, Jamshid Sabbaghzadeh^1^, Atefeh Ghaderi^1^, Shahram Solaymani^1,*^, Robert S. Matos^3^, Ștefan Țălu^4^, Henrique D. da Fonseca Filho^5^, Amir Hossein Sari^1^, Hanieh Kiani^6^,

Amir hossein Salehi shayegan^1,7^, Mahdi Astani Doudaran^1^

^1^Quantum Technologies Research Center (QTRC), Science and Research Branch, Islamic Azad University, Tehran, Iran.

^2^Physics Department, West Tehran Branch, Islamic Azad University, Tehran, Iran.

^3^Federal University of Amapá-UNIFAP, Amazonian Materials Group, Physics Department, Macapá, Amapá, Brazil.

^4^Technical University of Cluj-Napoca, The Directorate of Research, Development and Innovation Management (DMCDI), Cluj-Napoca, Cluj county, Romania.

^5^Federal University of Amazonas-UFAM, Laboratory of Synthesis of Nanomaterials and Nanoscopy, Physics Department, Manaus, Amazonas, Brazil*.*

^6^ Physics Department, Science and Research Branch, Islamic Azad University, Tehran, Iran.

^7^Mathematics Department, Faculty of Basic Science, Khatam-ol-Anbia (PBU) University, Tehran, Iran.

**Corresponding authors ***:

**Shahram Solaymani**

Quantum Technologies Research Center (QTRC), Science and Research Branch, Islamic Azad University, Tehran, Iran.

Phone: +989194947717, Fax: +982144805410

E-mail: [shahram22s2000@yahoo.com](mailto:shahram22s2000@yahoo.com) ; [shahram.solaymani@ipm.ir](mailto:shahram.solaymani@ipm.ir)

**Competing interests**

The authors declare no competing interests.

**Data availability statement:**

The datasets used and/or analyzed during the current study available from the corresponding author on reasonable request.

**APPENDIX**

1. Tauc relation

To plot with respect to the incident photon energy, we used the well-known Tauc relation [1]:

$(\alpha h\nu)^{\frac{1}{m}}=c(h\nu-E_{g})$ (1)

where C is a constant independent of the photon energy, α is the absorption coefficient, hν is the absorbed photon energy and E_g_ is the optical gap energy. The value of the exponent m denotes the nature of the transition; 1/2 for direct allowed, 3/2 for direct forbidden, 2 for indirect allowed, and 3 for indirect forbidden transitions [1].

1. Electrical properties

The I–V characteristics of the NiO/Si heterojunction device were determined by the well-known formula [2]

$I=I_{S}[\exp(\frac{q(V-IR_{S})}{nkT})-1]$ (2)

Here in, n, V, q, k, T, I_S_ and R_s_ are ideality factor, applied voltage, electronic charge, Boltzmann’s constant, absolute temperature, saturation current and series resistance, respectively.

Reverse saturation current I_S_ can be given by

 (3)

where A, A* and ϕ_b_ are the effective diode area, Richardson constant and diode barrier height values, respectively. The formula is given in Eq. (3) can be modified to obtain the barrier height value ϕ_b_ as

 (4)

The reverse saturation current I_S_ was determined by extrapolating semi-log I–V curve and intercepting the current axis at 0 V. Value of Richardson constant for Si is A*=32 A cm^−2^ K^−2^ [3]. In addition, another electrical parameter, the ideality factor, is calculated from the semi-log I–V curve by the following expression, derived from Eq. (2) [2].

$n=\frac{q}{kT}(\frac{dV}{d(\ln(I))})$ (5)

1. Cheung–Cheung’s method

In the present study, we used the method proposed by Cheung–Cheung’s [59] for determining series resistance as well as the other electrical parameters. Here, the R_S_ and n are calculated by using Eq. (5) and the slope of dV/dln (I) versus I graph in Fig. 15 which corresponds to the higher bias voltage region.

$\frac{dV}{d\ln(I)}=\frac{nkT}{q}+IR_{S}$ (6)

The R_S_ and ϕ_b_ values can also be calculated from Eq. (7) and the slope of the empirical H (I) versus I graph (in Fig. 15) obtained from the Cheung–Cheung’s equation, expressed in Eq. (8).

 (7)

and

$H(I)=V-(\frac{nkT}{q})\ln(\frac{1}{AA^{*}T^{2}})$ (8)

**References**

[1] Ilkhani M., Dejam L. Structural and optical properties of ZnO and Ni:ZnO thin films: the trace of post-annealing, J. Mater. Sci.: Mater. Electron., **32**, 3460–3474 (2021).

[2] Rhoederick E.H., Williams R.H. Metal-Semiconductor Contacts, Oxford University Press, Oxford, p. 54. (1988).

[3] Kaplan H.K., Sarsıcı S., Akay S.K., Ahmetoglu M. The characteristics of ZnS/Si heterojunction diode fabricated by thermionic vacuum arc, J. Alloys Compd., **724**, 543–548 (2017).
